# Artificial Intelligence Approaches for Predicting the Risks of Durable Mechanical Circulatory Support Therapy and Cardiac Transplantation

**DOI:** 10.3390/jcm13072076

**Published:** 2024-04-03

**Authors:** Chloe Grzyb, Dongping Du, Nandini Nair

**Affiliations:** 1 PennState College of Medicine, Heart and Vascular Institute, Milton S. Hershey Medical Center, 500 University Dr, Hershey, PA 17033, USA; cgrzyb@pennstatehealth.psu.edu; 2 Department of Industrial and Structural Engineering, Texas Tech University, Lubbock, TX 79409, USA; dongping.du@ttu.edu

**Keywords:** AI-driven risk prediction models

## Abstract

**Background:** The use of AI-driven technologies in probing big data to generate better risk prediction models has been an ongoing and expanding area of investigation. The AI-driven models may perform better as compared to linear models; however, more investigations are needed in this area to refine their predictability and applicability to the field of durable MCS and cardiac transplantation. **Methods:** A literature review was carried out using Google Scholar/PubMed from 2000 to 2023. **Results:** This review defines the knowledge gaps and describes different AI-driven approaches that may be used to further our understanding. **Conclusions:** The limitations of current models are due to missing data, data imbalances, and the uneven distribution of variables in the datasets from which the models are derived. There is an urgent need for predictive models that can integrate a large number of clinical variables from multicenter data to account for the variability in patient characteristics that influence patient selection, outcomes, and survival for both durable MCS and HT; this may be fulfilled by AI-driven risk prediction models.

## 1. Introduction

Artificial intelligence (AI) has the potential to improve risk prediction in cardiovascular medicine through the integration and analysis of complex clinical data. In recent years, an expanding body of literature has studied the applications of AI to predict the outcomes of patients undergoing durable mechanical circulatory support (MCS) and heart transplantation (HT) for end-stage heart failure. Durable MCS can be a bridge to transplantation or a long-term solution, while HT provides a lifesaving surgical therapy, although it is limited by the availability of donor organs. The outcomes of each of these therapies are dependent on an intricate and dynamic array of variables, such as comorbidities, laboratory values, echocardiogram findings, and biomarkers. In addition to the prognostication of perioperative risks, AI risk prediction models can analyze the preoperative variables to select the optimal type of patient for durable MCS or HT in order to maximize resource allocation and minimize complications such as mortality [1,2].

While technological advances have improved the outcomes for durable MCS and HT, patients continue to develop complications. Cerebrovascular complications, right ventricle (RV) failure, and gastrointestinal (GI) bleeding are common complications in patients receiving durable mechanical support. A stroke or transient ischemic attack (TIA) can occur anytime during the post-implant period and beyond. Complications unique to HT include primary graft dysfunction, allograft rejection, and graft vascular disease, developing weeks to years after transplantation. Chronic immunosuppression is critical to transplant success but also remains a leading cause of complications like malignancy and infection. AI risk prediction models have improved in their ability to predict post-operative complications but remain limited in clinical settings due to missing data and class imbalances [3,4,5]. AI models based on the Bayesian Network, which utilize hemodynamic, clinical, and demographic data, as well as others based on Active Network Management (ANM) image recognition, are described in this review. It is important to note that AI is an up-and-coming area in assessing risk prediction but needs more investigations to be useful as a bench-to-bedside tool. Ethical, logistical, and legal aspects as well as algorithm bias and usefulness in enhancing healthcare outcomes still need to be standardized. 

Additionally, the external validation of prospective studies will be required for broad applications [6,7,8].

Complications post-durable MCS implant and post-heart transplant pose different and unique challenges. 

Complications post-durable MCS as a bridge to transplantation have been accompanied by significant early and late complications post-implant. Stroke occurs with continuous-flow pumps and pulsatile devices. The stroke risk is highest in the first three months after implant, but contradictory evidence has shown bimodal distributions at the 9- to 12-month mark [9]. The risk of both hemorrhagic and ischemic strokes is high and estimated at 10–30% within 2 years of device implantation [10,11]. Embolic strokes appear more common than hemorrhagic strokes with all device designs and may arise not only from clot formation in situ in the pump, but also from ingested thrombi propelled through the device. The type of device also influences the stroke incidence [12,13]. Clots formed in the in-flow and out-flow grafts can also cause heart failure due to pump dysfunction.

Approximately 16% of durable MCS patients suffer at least one post-operative stroke [14]. The risk factors associated with stroke include treatment-related factors, including specific devices, prior cardiac surgery, and patient-related factors. There remains a need for long-term data and a more accurate characterization of the natural history of post-implant strokes [13,14]. 

A device-related infection can be an early or late complication of durable MCS. Pump-related infections involve the driveline, pump pocket if it is an axial pump, or other components of the pump itself. The pump design, patient susceptibility, surgical technique, causative organism, and driveline care appear to be the primary risk factors of infection [15].

RV failure is an important and common complication of durable MCS, affecting between 20–50% of LVAD patients [16]. Survival at one year after a durable MCS implant is approximately 83% [17]. Right heart failure after an LVAD implant increases the risk of death and prolongs the length of hospitalization [18]. Although survival is influenced by multiple factors, younger and less sick patients at the time of LVAD implantation tend to have better outcomes.

The neurological complications post-durable MCS can be devastating. Further investigations into risk factor identification and prevention are warranted [19]. 

Interestingly, the newer pumps, which are magnetically levitated, have a lower rate of complications in the 5-year data as per the 14th annual INTERMACS report. GI bleeding, stroke, and device malfunction/pump thrombus have significantly decreased [20].

Complications post-heart transplant include primary graft dysfunction (PGD), stroke, and acute cellular and acute antibody-mediated rejection. A 2018 meta-analysis demonstrates that the recipient age, congenital etiology, creatinine levels, pulsatile flow, durable MCS status, donor age, and female donor-to-male recipient sex mismatch influence the 1-year mortality status post-heart transplant [21]. Primary graft dysfunction post-transplant (PGD) is one of the leading causes of early mortality in HT, with a prevalence of 2.3% to 28.2% [22,23,24,25,26,27,28]. The risk factors for the development of PGD have been identified as advanced donor and recipient age, ischemia time, pre-transplant recipient diabetes, hemodialysis, African American race, recipient amiodarone treatment, higher pre-transplantation right atrial pressure, and donor ischemia time [26,27]. Further investigation into the underlying causes and management of PGD is critical in halting the increased incidence.

Cerebrovascular/neurological complications after cardiac transplant in the early post-transplant period are possible [29]. More recent data on the neurological complications post-heart transplant estimate an incidence of 3–10%. Additionally, ischemic strokes have an incidence of 2.5%, while transient ischemic attacks (TIAs) have been estimated to occur up to 13% post-heart transplantation [29,30]. Perioperative cerebrovascular complications are more common after a cardiac transplant than other cardiac surgical procedures. 

Antibody-mediated rejection (AMR) is another major risk factor for mortality, as well as allograft injuries, including systolic dysfunction, restrictive physiology, and cardiac allograft vasculopathy (CAV) [31]. AMR may present as early as days to years after the transplant [32]. Earlier antibody rejection is associated with preformed HLA antibodies, whereas rejection occurring months to years after the transplant is due to the development of de novo antibodies. HLA antibodies and immune complexes are commonly found in patients exhibiting rejection. The patients with an elevated risk were identified as female, multiparous, high panel-reactive antibody, CMV seropositivity, those receiving a second transplant, OKT3 induction therapy, and positive crossmatch [32]. 

Cardiac transplantation is still the gold standard of treatment for patients who have end-stage heart failure. Advances in immunosuppression have contributed to better long-term outcomes even in older and higher-risk recipients. Heart transplant recipients currently have a median survival of 12–13 years [33]. One of the important causes of early mortality is primary graft failure. Factors that influence late mortality include malignancy, chronic rejection, and cardiac allograft vasculopathy (CAV). CAV is the single most significant long-term complication of heart transplantation. Concentric hyperplastic lesions comprised of smooth muscle cells and matrix build-up on the intimal layer of the coronary arteries are the anatomical highlights of this disease process. CAV diffusely affects allograft vessels, resulting in narrowing, decreased perfusion, ischemia, and graft failure. Strategies for the maintenance of graft patency focus on immunomodulation, anti-thrombogenesis, as well as the modulation of endothelial progenitor cell and endothelial cell adhesion, proliferation, and activation [32]. This is an aggressive disease that is unresponsive to existing therapies such as immunosuppression modifications [34]. A deeper understanding of the mechanisms and risk factors underlying CAV is imperative to the optimal management of this complication. 

The key areas of knowledge gaps are in patient selection and avoiding complications post-durable LVAD implantation and/or cardiac transplantation. Further investigations into the risk factors should focus on elucidating the characteristics of the recipient patient and donor allograft. In patients receiving durable MCS, the prevention of strokes is an important aspect influencing the outcomes. Such knowledge gaps may be closed using AI-driven technology with big data.

## 2. Materials and Methods

This is a qualitative review. Hence, a literature search was conducted on PubMed and Google Scholar. All published papers on risk prediction in mechanical circulatory support and cardiac transplantation were reviewed qualitatively, and the information was synthesized to arrive at the results and conclusions presented in this review. The keywords used were risk prediction models, linear risk prediction/AI-driven models for risk prediction in durable MCS and heart transplantation, prediction of outcomes in heart transplants, complications of heart transplants, and durable MCS.

## 3. Results

### 3.1. Linear Risk Prediction Models and Their Limitations for Durable MCS

Two popular clinical RV failure risk scores used for predicting the post-operative RV failure are the Penn and CRITT scores [18,35]. These models were put forth to predict RV failure post-LVAD implantation. These incorporate the hemodynamic values, qualitative assessments, and laboratory values. The CRITT model offers a five-variable risk calculator to determine the suitability for uni- or bi-ventricular support. The negative predictive value was 93% and had an AUC of 0.80 ± 0.04. The Penn model found the most significant predictors for the need for an RVAD, which were the RV stroke work index, cardiac index, severe pre-operative RV dysfunction, prior cardiac surgery, systolic blood pressure, and creatinine levels. This algorithm predicts which LVAD patients will require an RVAD with a >80% sensitivity and specificity. The Destination Therapy Risk Score was an early model that was later found to be poorly predictive in continuous-flow VAD patients [36,37]. The HeartMate II Risk Score (HRMS) was designed to predict the 90-day mortality in patients undergoing LVAD implantation and factored in the age, serum creatinine, international normalized ratio, albumin levels, and center volume. The HMRS was found to correlate with short- term and long-term post-LVAD survival and could identify patients with a low (8%), medium (12%), and high (26–32%) risk of 90-day mortality (c-statistic 0.7 and 0.71) [38,39]. Soliman et al. developed an effective linear regression model for right heart failure after durable MCS implantation [40].

However, linear regression models would need further development and testing to assess their efficacy in comparison to AI-driven models. The risk models derived from logistic regression studies are summarized in Table 1A.

### 3.2. AI Models for Durable MCS and Their Limitations

AI risk prediction models for durable MCS exist at this time, all of which have their strengths and limitations. The Cardiac Outcomes Risk Assessment (CORA) model is the earliest model, which risk-stratified patients using Bayesian networks to predict short- and long-term LVAD mortality [41]. The variables comprised of 226 clinical, demographic, hemodynamic, and laboratory values, social characteristics, and functionalities [41]. Bayesian classification models were developed to predict mortality at five endpoints post-implant, achieving c-statistics of 0.91, 0.82, 0.82, and 0.81 for predicting mortality at 30 days, 90 days, 6 months, 1 year, and 2 years after implantation. The prediction of mortality at different periods is influenced by the risk factors used in developing the risk prediction model. Hence, when more risk factors are applied, the mortality of a given patient population will increase. This demonstrates that identifying key risk factors is the most important aspect of building risk prediction models.

Kanwar et al. developed a model that predicts survival in durable LVADs. Their 2018 model predicts survival at 1, 3, and 12 months after LVAD implantation using INTERMACS data [42]. Their Bayesian network analysis showed accuracies between 76% and 87% and c-statistics of 0.7–0.71 for the 1-, 3-, and 12-month mortality. The variables predicting 1-month mortality (number of acute events 48 h before surgery, temporary MCS, and renal and hepatic dysfunction) differed from those predicting 12-month mortality (advanced age, frailty, device strategy, and chronic renal disease).

In 2021, Kilic et al. used INTERMACS data to predict the 90-day and 1-year mortality using ML and compared the results to an equivalent logistic regression model. The XGboost model had a max AUC of 0.74. They found a substantial improvement in the 90-day (logistic regression 0.536 versus 0.752 with AI model) and 1-year (logistic regression 0.555 versus 0.726) mortality [2]. To date, the limited ML models of mortality demonstrate superiority over traditional linear models (c-statistic between 0.7–0.81) compared with logistic regression models of mortality (c-statistics range from 0.54–0.55) [18,42,43,44,45,46].

Kanwar et al. 2016 developed a model which predicts right ventricular failure using INTERMACs data [5]. They developed acute, early, and late right ventricular failure models that included preoperative variables (from demographics, laboratory values, hemodynamics, and medications), respectively. This model revealed c-statistics of 0.9, 0.83, and 0.88 for acute, early, and late RVF, respectively. These were superior to the prior linear models, with c-statistics ranging from 0.55–0.65 [18,42,43,44,45,46]. Missing data from the INTERMACS registry limited these studies. Additionally, the skewness of the data towards the absence of RVF affected the effectiveness of the models in predicting RVF failure despite the promising c-statistics. 

A model from Shad et al. predicts post-operative RV failure, which they developed using a 3D convolutional neural network with preoperative transthoracic echocardiograms as input. The model achieved a c-statistic of 0.729, outperforming a team of human experts on the same task evaluation, and was compared to the CRITT and Penn linear models [4]. Saliency maps were utilized for model interpretation, which identify motion characteristics in specific regions of the heart that contribute to the prediction.

The limitations of models using INTERMACS data are the prevalence of missing data and severe class imbalances within the registries. Furthermore, the INTERMACS data used at those times primarily included patients with Heart Mate II, which makes the data less generalizable across the entire spectrum of devices. Other limitations of the dataset analyses are their retrospective nature, with the uneven distribution of continuous and categorical variables [47,48]. 

Through the use of imaging and spectral data, Misumi et al. developed an AI-trained model using the acoustic spectra of LVAD devices as input to predict cerebrovascular accidents and aortic valve insufficiency. Their novel methodology predicts cerebrovascular accidents with an accuracy of 0.98 [47,48]. The prediction of aortic valve insufficiency in LVAD patients had an accuracy of 91% with an ROC of 0.73 [48]. Promising Active Network Management (ANM)-based image recognition models have been used to predict infection severity identification on LVAD driveline exit-site images with a 67% accuracy, which is close to expert-level performance [49]. Risk-stratification models for durable MCS reveal an array of risk factors including demographic, clinical, and hemodynamic data, which attempt to predict RV failure and the overall survival, reflecting the multifactorial approach to decision making in clinical practice. A list of selected AI-driven studies is shown in Table 1B. Most recently published is the STOP-RVF study, which generates a risk assessment tool for the prediction of right ventricular failure and consequent mortality [50]. This is a supervised machine learning model with a c-statistic of 0.73–0.75.

### 3.3. Linear Risk Prediction Models for HT

Models from Aaronson et al. integrate 80 clinical characteristics from 268 patients with advanced heart failure to predict the 1-year post-transplant survival with a c-index of 0.61 [51]. A notable model is the IMPACT score, which uses a 50-point index to predict the 1-year post-transplant mortality with a c-statistic of 0.65 [52]. Segovia et al. developed a RADIAL score, which showed a good ability to predict the development of PGD and could be useful in the prevention and prompt treatment of this complication [24]. Risk factors of this model included an age over 60, diabetes mellitus of the recipient, right atrial pressure greater than 10, donor age over 30 years, and ischemic time > 4 h. The risk stratification score (RSS) model found that pre-transplant recipient variables influence early and late graft failure. The strongest negative predictors of 1-year graft failure were RVAD only, ECMO, renal failure, LVAD, total artificial heart, and advanced age. The 1-year survival for the low-risk, intermediate-risk, moderate-risk, elevated-risk, and high-risk groups were 93.8, 89.2, 81.3, 67, and 47%, respectively [53].

Weiss et al. developed a donor risk index (DRI) that predicts the short- and long-term mortality. It is a 15-point scoring system that incorporates the ischemic time, donor age, race mismatching, and BUN/creatinine ratio. Each point increases the risk of 1-year death by 9%. It also predicted the 30-day mortality (OR = 0.11 [1.08 to 0.14], *p* < 0.001) [54]. Selected logistic regression studies are shown in Table 2. There is a need for AI models that focus not only on survival but also on long- and short-term rejection phenomena, as well as immunosuppression and subsequent cancer development. These models remain limited in their clinical use due to the poor predictive powers of outcomes that are multifactorial.

### 3.4. AI Models of HT

The applications of AI can be separated into three areas of application: mortality, graft failure, and outcomes in HT [55]. Most of the evidence focuses on the use of ML models in predicting mortality and survival post-transplant. Twelve ML models were developed for predicting waitlist mortality and post-transplant rejection [1,24,52,53,54,55,56,57,58,59,60,61,62,63]. Medved et al. implemented a two-layer neural network model to predict the waitlist outcomes, such as still waiting, transplanted, or deceased 180 days, 365 days, and 730 days after being listed. Their model achieved F1 macro scores of 0.674, 0.680, and 0.680 at these three time points. Additionally, Medved et al. created an organ allocation policy using a neural network algorithm. The simulation study suggested that the neural network policy extends the mean survival to 4700 days, compared to the mean survival of 4300 days under the wait time policy or the clinical rules policy [64,65].

Yoon et al. developed a survival model using decision tree techniques to predict post-transplant survival. The approach was to use clusters of predictors called “trees of predictors” (ToPs). This technique used clinical data to discriminate the differences in the survival of patients at different time points after LVAD implantation. The inputs ranged from clinical to demographic features of the recipient, donor, and donor–recipient compatibility attributes. The inputs included the age, gender, prior transplantation, transfusions, hepatic and renal functions, ventilator assistance, HLA mismatch, and blood type. Their model achieved a c-statistic of 0.66 for the 3-month survival prediction and a sensitivity of 0.8 for the 3-year survival prediction [56].

Miller et al. compared a neural network model with traditional regression models to predict the 1-year survival after pediatric heart transplantation. The input of variables included donor and recipient characteristics such as the age, gender, race, weight, ABO blood type, diagnosis, payor type, creatinine level pre-transplant, days on waitlist, medical condition, presence of defibrillator/LVAD/ECMO at transplant, mechanical ventilation, inotropic agents, donor blood type, and recipient blood match [57].

The neural network demonstrated the highest c-statistic of 0.66, though only marginally superior to logistic, ridge, and LASSO regression models. The study highlighted the crucial role of data quality in achieving accurate prognoses using ML techniques. In a subsequent study, a random forest model yielded c-statistics of 0.72, 0.61, and 0.60 for the 1-, 3-, and 5-year mortality, respectively, with a poor sensitivity (0.07–0.49), which was attributed to missing data and data imbalance issues [58].

Furthermore, Kampaktsis et al. reported c-statistics of 0.689, 0.642, 0.649, 0.637, and 0.526 for Adaboost, logistic regression, decision tree, support vector machine, and K-nearest neighbor models, respectively, in predicting the 1-year mortality after heart transplant [59]. All the studies utilized the UNOS data registry, and the ML models developed converged to a similar performance. A key takeaway is that ML models outperform conventional risk scores, such as the Index for Mortality Prediction After Cardiac Transplantation (IMPACT) score [52]. Ahady Dolatsara et al. designed a two-stage machine learning (ML) framework. In the first stage, an ML model is employed to predict the transplant outcomes at different periods of interest. Subsequently, an isotonic regression is performed in the second stage to calibrate the survival probability. Their framework yields c-statistics ranging between 0.6 and 0.7 for predicting survival from 1 to 10 years after heart transplantation [60]. Ayers et al. combined multiple machine-learning models, including logistic regression, deep neural network, random forest, and Adaboost, into an ensemble model. The ensemble model demonstrated an improved c-statistic of 0.764, outperforming each model, with c-statistics ranging from 0.649 to 0.691 for predicting the 1-year mortality. This is the first ensemble ML model developed for predicting heart transplantation outcomes [61]. Beyond the registry study, Zhou et al. assessed the efficacy of random forest and gradient-boosting machine in predicting the 1-year mortality using a single-center dataset. The random forest model yielded a higher c-statistic (0.801), whereas the gradient boosting machine demonstrated a superior sensitivity (0.271), indicating its better capability in predicting the outcomes of the minority (deceased) group [62]. However, as highlighted in other studies, the low sensitivity suggests room for improvement, particularly for potential clinical implementation. Nilsson et al. developed a non-linear artificial neural network survival model with a concordance index of 0.6 and a c-statistic of 0.65 for predicting the 1-year mortality [63]. This outperforms existing scoring models, including the donor risk index (DRI), risk-stratification score (RSS), and IMPACT score. Additionally, they created a decision tree model to interpret the results by assessing the impacts of recipient–donor variables on survival over time. Predictive models of graft failure and mortality were found to be more accurate than traditional linear models. Major predictors of graft failure and mortality were immunosuppressive regimens, recipient age, organ ischemia time, length of hospital stay, and congenital heart disease [1,3,63,64,65]. ML models of mortality and graft patency revealed new risk factors that were not previously identified in linear models, such as length of stay. Further studies are needed to clarify the variables influencing the post-operative outcomes and mortality. Recipient age was found to be significant in both linear and AI models. Predictive power was improved for patients over 60, reflecting the age group most represented in existing datasets. The variables prevalent in other populations, such as congenital heart disease (CHD) were unaccounted for in all but one model [66]. Datasets should incorporate detailed data from all age groups or stratify their model according to the age group. Prolonged ischemic time is a known factor predicting graft failure and mortality at 1 year more so than at 5 years, as most failure due to ischemia occurs early post-operatively. Current AI models identified the immunosuppressive regimen as more important in the prediction of graft failure than mortality [1,27]. However, few databases have granular immunosuppression regimen data, which may limit the strength of this association.

**Table 1:** (A) Selected risk prediction models using logistic regression-driven statistical models for MCS. (B) Selected risk prediction models using AI-driven statistical methods for MCS.

**(A)**					
**Study (Authors and Reference #)**	**Study Type**	**Study Method**	**Subjects**	**Duration (Months)**	**Conclusion of Study**
Cowger et al., 2013 [38]	Multicenter, prospective	Logistic regression, HMII Risk Score	1122 patients enrolled into HMII bridge to transplantation and destination therapy trials	3-month mortality	Stratifies mortality risk in HMII candidates; AUC 0.71, 95% CI: 0.66–0.75.
Atluri et al., 2013 [35]	Retrospective, single center	Multivariable logistic regression, CRITT score	218 patients who underwent VAD implant: LVAD = 167, BIVAD = 51	Patients between 2003 and 2011	5-variable risk stratification score to determine suitability for uni- or bi- ventricular support; NPV 93%, AUC: 0.80 ± 0.04.
Fitzpatrick et al., 2008 [18]	Retrospective, single center	Logistic regression, PENN score	266 LVAD recipients	Patients between 1995 and 2007	Most significant predictors for RVAD need were creatinine level, prior cardiac surgery, systolic blood pressure, stroke work index, severe pre-operative RV dysfunction; showed >80% sensitivity and specificity.
**(B)**					
**Study (Authors and Reference #)**	**Study Type**	**Study Method**	**Subjects**	**Duration (Months)**	**Conclusion of Study**
Kilic et al., 2021 [2]	Retrospective, multicenter	Extreme gradient: XG Boost and logistic regression	Adults aged 19 years or older undergoing primary durable LVAD implantation as part of the INTERMACS database (16,120)	3 and 12 months	ML was associated with a statistically significant improvement in discriminatory performance for both 90-day and 1-year mortality; ML can be used independently and as an adjunct to logistic regression.
Kanwar et al., 2018 [42]	Retrospective, multicenter	Bayesian models, Cardiac Outcomes Risk Assessment (CORA)	Adults over 18 who received an initial primary continuous flow LVAD or LVAD and right ventricular assist device (RVAD) in combination (10,277)	1, 3, and 12 months	Accuracy of all Bayesian models was between 76% and 87%, with an area under the receiver operative characteristics curve between 0.70 and 0.71.
Shad et al., 2021 [4]	Retrospective, 3 contributing centers	Three-dimensional convolutional neural network, built using the Keras framework with a TensorFlow 2.1 backend and Python	18 years or older with at least one pre-operative transthoracic echocardiogram undergoing LVAD placement (941)	Implant to MCS-ARC definition of post-operative RV failure	A video AI system trained to predict post-operative RVF in the setting of MCS can outperform human experts on the same task evaluation (AUC 0.729).
Loghmanpour et al., 2016 [5]	Retrospective, multicenter	Bayesian models, CORA models	Continuous flow LVAD as the primary implant and age ≥18 years (10,909)	Acute (<48 h), early (48 h to 14 days), and late (>14 days)	Three separate Bayesian models for acute, early, and late RVF substantially outperformed the existing linear risk scores in their ability to predict the risk of RV failure.
Loghmanpour et al., 2015 [41]	Retrospective, multicenter	Bayesian models, CORA models	Continuous flow LVAD patients over 19 years (8050)	1, 3, 6, 12, and 24 months	Bayesian models predicting mortality at 5 time points out performed HeartMate II Risk Score (HMRS); preimplant interventions, ECMO, and ventilators were major risk factors.
Misumi et al., 2019 [47]	Retrospective, single center	ML	Acoustic spectra from 4 patients with HeartMate II CF-LVAD who developed CVA during 1-year follow -up; 81 sound signals from 4 patients	12 months	ML model predicted cerebrovascular accident in patients with a VAD using acoustic spectra with AUC 0.98, F-measure 0.89.
Misumi et al., 2021 [48]	Prospective, single center	ML	Acoustic spectra from 13 adults with Jarvik2000 LVAD; 245 spectra from 13 patients	24 months	ML trained on acoustic spectra offers a novel modality for prediction of aortic regurgitation in LVAD patients.

**Table 2:** Selected risk prediction models using logistic regression-driven statistical methods for the transplant population.

Study (Authors and Reference #)	Study Type	Study Method	Subjects	Duration (Months)	Conclusion of Study
Hong et al., 2011 [53]	Multicenter (UNOS), retrospective;	Multivariable logistic regression	11,703	12-month graft failure	The risk stratification score (RSS) model found that pre-transplant recipient variables influence early and late graft failure; the strongest negative predictors of 1-year graft failure were RVAD only, ECMO, renal failure, LVAD, total artificial heart, and advanced age; the 1-year survival for the low risk, intermediate risk, moderate risk, elevated risk, and high-risk groups were 93.8, 89.2, 81.3, 67, and 47%, respectively.
Aaronson et al., 1997 [51]	Single center, prognostic	Multivariable proportional hazard survival models	286 patients with advanced heart failure	12-month survival	Determined 1-year survival in low-risk (93, 88%), medium-risk (72, 60%), and high-risk (43, 35%) patients; Medium- and high-risk patients are likely to die or require transplantation within one year; transplantation can be deferred in the low-risk group.
Weiss et al., 2012 [54]	Multicenter (UNOS), retrospective	Multivariate logistic regression	22,252	12-month survival	The donor risk index (DRI) model is a 15-point scoring system incorporating ischemic time, donor age, race mismatching, and BUN/creatinine ratio; each point increases the risk of 1-year death by 9%; it also predicted the 30-day mortality (OR = 0.11 [1.08 to 0.14], *p* < 0.001).
Weiss et al., 2011 [52]	Multicenter, prospective	Multivariable logistic regression	21,378	12-month mortality post-transplant	A 50-point IMPACT scoring system incorporated 12 recipient-specific variables to accurately predict the mortality, with a c-statistic of 0.65.
Segovia et al., 2011 [24]	Single center, prospective	Multivariate stepwise logistic regression model	621	Post-transplant	6 multivariate risk factors of PGF (RA pressure >10 mmHg, recipient age >60, diabetes, inotrope dependence, donor age >30 years, and ischemic time >40 min: RADIAL); rates of actual and predicted PCG incidence showed good correlation (c-statistic 0.74).

## 4. Discussion

### 4.1. Superiority of AI and ML Predictive Models

ML models identify non-linear relationships and integrate an array of variables to predict patient outcomes. ML models are advantageous over traditional regression models, because they can analyze data in complex structures to create new, dynamic relationships and determine the variables most influential on a particular outcome. Traditional linear models determine the probability of an event occurring due to the influence of a specific variable. ML allows for a model to be built based on the input of an array of variables and tailors to the model based on the most influential factors. This review reveals that preliminary ML models predicting LVAD failure and mortality exhibit superiority over traditional logistic regression models. In HT, AI models of graft failure and mortality were found to be more accurate than traditional linear models. This has the potential to determine the candidacy for HT and durable MCS through the prediction of post-operative outcomes. In addition, patients at risk of end-stage heart failure may also be identified earlier and referred to a life-prolonging therapy before disease progression, which renders them ineligible for surgery. Optimized models offer a helpful adjunct to clinical decision making. A list of selected studies is shown in Table 3A,B.

AI-based models may perform better than those developed using traditional statistics, but the risk of bias, the need for external validation, and lesser-known applicability at this time may affect AI-based tools. More investigations are needed using large, high-quality databases for AI-driven models to become a mainstay [67,68,69].

### 4.2. Future Directions for AI Risk Prediction

The main barrier to improved AI risk prediction for both durable MCS and CT is the need for higher quality, complete, and accurate data from a diverse population to input into datasets. Current datasets, such as INTERMACS, would benefit from validation and standardization procedures to improve the quality and availability of the clinical data. As transplant and durable MCS implants occur relatively frequently, the entered data must be granular and error-free. For example, most databases do not consistently collect granular data regarding immunosuppressive regimens, which were identified as a predictive variable in graft failure and mortality. AI models often require extensive clinical data to achieve a satisfactory performance. Smaller databases inherently encourage overfitting to inaccurate data points and result in less generalizable analyses. Broader data collection may be considered by registries to optimize ML prediction. The ability to interpret ML and AI models needs to improve to broaden their applications in clinical practice. Although these models demonstrate a superior performance compared to traditional regression analysis, the majority of them operate as black boxes, making it challenging to trace and explicitly articulate the logic behind risk computations. Enhancing the interpretability of ML/AI models is important for deriving clinical implications from risk predictions.

**Figure 1:**
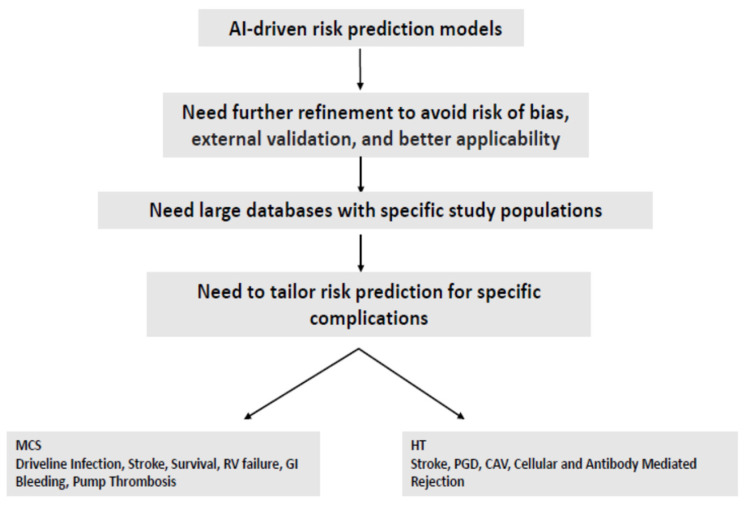
Future Perspectives.

The cost and staffing remain barriers to optimal data collection. Prospective registries eliminate the bias associated with current retrospective datasets, offering a solution to current data issues, but at a significant cost. Once reliable and accurate models are developed, their accessibility and ease of use by clinicians will be critical in ensuring that the models are used in the real world. This could involve their incorporation into electronic medical records, smartphone apps, and web-based sites. Finally, AI and ML models should be developed to predict additional post-operative outcomes. Many models for durable MCS focus on RV failure and survival. The current models of durable MCS do not address additional significant complications of durable MCS, including driveline infections, cerebrovascular events, and bleeding. The AI models did not address significant complications such as graft vascular disease and stroke but could further explore the predicted roles of immunosuppressive regimens. Future AI models should further investigate additional adverse events for a more accurate prediction of post-implantation outcomes. Figure 1 summarizes the future perspectives on using AI technology for risk prediction.

## 5. Conclusions

Currently, ML model implementation in durable MCS and HT reveals superiority over linear models in predicting the endpoints of each. The existing models of durable MCS and HT primarily focus on mortality. Additional models of durable MCS predict RV failure, cerebrovascular accidents, and aortic insufficiency. The models of HT predict waitlist mortality and pre-transplant rejection. The main limitations of the current models are due to missing data, data imbalances, and the uneven distribution of variables in the datasets from which the models are derived. There is an urgent need for predictive models that can integrate a large number of clinical variables from multicenter data to account for the variability in patient characteristics that influence the patient outcomes and survival for both durable MCS and HT. 

## Figures and Tables

**Table 3:** Selected risk prediction models using AI-driven statistical methods for HT.

**(A)**					
**Study (Authors and Date)**	**Study Type**	**Study Method**	**Subjects**	**Duration (Months)**	**Conclusion of Study**
Kampaktsis et al., 2021 [59]	Multicenter, retrospective	LR, decision tree, K-nearest neighbor, logistic regression, Adaboost	18,625	12-month mortality post-transplant	Reasonable predictive accuracy of mortality after transplant; highest predictive performance with Adaboost model; AUCs for the prediction of 1-year survival were 0.689, 0.642, 0.649, 0.637, and 0.526 for the Adaboost, logistic regression, decision tree, support vector machine, and K-nearest neighbor models, respectively.
Zhou et al., 2021 [62]	Single center, retrospective	Artificial neural network, gradient boost machines, Adaboost, random forest, support vector machine, logistic regression	381	12-month mortality post-transplant	Random forest plot performed highest discrimination with largest AUG (0.801) when validated; albumin level, recipient age, and left atrium diameter were the most important prognostic variables.
Ayers et al., 2021 [61]	Multicenter (UNOS), retrospective	Deep neural network, logistic regression, Adaboost	33,657	12-month mortality post-transplant	Model derived from preoperative variables; final ensemble ML model outperformed traditional models (*p* < 0.001); AUROC of logistic regression (0.649) vs. random forest (0.691), deep neural network (0.691), Adaboost (0.653), and final ensemble ML (0.764).
Ahady Dolatsara et al., 2020 [60]	Multicenter (UNOS), retrospective	Logistic regression, XG Boost, linear discriminant analysis, random forest, artificial neural network, classification and regression tree	103,570	First, ML was used to predict transplant outcomes for time periods; second, survival probabilities were calibrated over time using isotonic regression.	First stage showed AUC (0.60 and 0.71) for years 1–10; the 10-year AUC of 0.70 is higher than most results; isotonic regression can calibrate survival probabilities for each patient over a 10-year period.
Agasthi et al., 2020 [1]	Multicenter (ISHLT registry), retrospective	Gradient boost machines	15,236	5-year mortality and graft failure	Length of stay, recipient and donor age, recipient and donor BMI, and ischemic time had the highest prediction of mortality; model used 87 variables to predict mortality and graft failure; AUC for 5-year mortality was 0.717 and 0.716.
Hsich et al., 2019 [66]	Multicenter (Scientific Registry of Transplant Recipients), retrospective	Random survival forest	33,069	NA	Identified strong and weak predictive variables from registry between 1985 and 2015; most predictive variables are currently in the tiered allocation system; new variables identified were eGFR and serum albumin.
Miller et al., 2019 [57]	Multicenter (UNOS), retrospective	Logistic regression, decision tree, neural networks, random forest, support vector machine	56,447	1-year mortality after transplant	ML did not result in improvements in 1-year prediction compared to traditional models (c-statistic 0.66 for all models); identified predictive variables consistent with prior findings, including age, renal function, liver function tests, hemodynamics, and BMI.
Miller et al., 2019 [58]	Multicenter (UNOS), retrospective	Artificial neural network, classification and regression tree, random forest	2802	1-, 3-, and 5-year mortality after pediatric transplantation	ML algorithms demonstrated a fair predictive ability but had a poor sensitivity; incomplete and missing registry data limit prediction; AUCs for 1-, 3-, and 5-year mortality were 0.72, 0.61, and 0.60, respectively.
**(B)**					
**Study Authors and Reference #**	**Study Type**	**Study Method**	**Subjects**	**Duration (Months)**	**Conclusions of the Study**
Yoon et al., 2018 [56]	Multicenter (UNOS), retrospective	Trees of predictors	95,275	1-, 3-month, and 10-year mortality	ToP improves survival prediction both post- and pre-transplant and performs better than existing clinical models and other ML methods; AUC for 3 months was 0.660 and best clinical risk score was 0.587; ToPs is practical and adaptable to clinical practice.
Medved et al., 2018 [65]	Multicenter (UNOS), retrospective	International Heart Transplantation Survival Algorithm (IHTSA), Index for Mortality Prediction After Cardiac Transplantation (IMPACT)	27,705	Compares IHTSA and IMPACT models in prediction of short- and long-term mortality after transplant	IHTSA showed better discriminatory power at 1 year and overall survival; IHTSA was more accurate than the IMPACT model; c-index for IHTSA was 0.627 and for IMPACT was 0.608.
Medved et al., 2018 [3]	Multicenter (UNOS), retrospective	International Heart Transplantation Survival Algorithm (IHTSA) and Lund deep learning transplant algorithm (LuDeLTA)	49,566	Predicts status of patients on list and post-transplant survival	The predicted mean survival for allocation according to the wait time was 4300 days, with clinical rules was 4300 days, and using neural networks was 4700 days.
Medved et al., 2017 [64]	Multicenter (UNOS), retrospective	Artificial neural network, Keras framework	27,444	180, 365, and 720 days after entering heart transplant list (outcome: waiting, transplanted, or dead)	Identified top ten weighted parameters affecting the outcome.
Nilsson et al., 2015 [63]	Multicenter (ISHLT registry), retrospective	Flexible nonlinear artificial neural network model (IHTSA)	56,625	1-, 5-, and 10-year survival	The IHTSA model can predict short- and long-term morality with a high accuracy (ROC 0.650); recipients matched to donors under 38 years had an additional survival of 2.8 years; model accuracy was excellent (0.6) at 1-, 5-, and 10-year survival.

## Data Availability

The data presented here are analyzed from the public domain and can be shared on request.
